# Assessment of Relative Asthma Risk in Populations Living near Incineration Facilities in Seoul, Korea

**DOI:** 10.3390/ijerph17207448

**Published:** 2020-10-13

**Authors:** Hyun-Joo Bae, Jung Eun Kang, Yu-Ra Lim

**Affiliations:** 1 Climate, Air Quality and Safety Research Group, Korea Environment Institute, Bldg B, 370 Sicheong-daero, Sejongsi 30147, Korea; hjbae@kei.re.kr; 2 Department of Urban Planning and Engineering, Pusan National University, 2 Busandaehak-ro63, Geumjeong-gu, Busan 46241, Korea; 3 Institute of Environmental Medicine, Seoul National University Medical Research Center, 103 Daehak-ro, Jongno-gu, Seoul 03080, Korea; yrlim326@gmail.com

**Keywords:** incineration plant, asthma, disease mapping, spatial relationship, rapid inquiry facility

## Abstract

While incineration is among the most commonly used technologies for waste disposal, there is ongoing public concern regarding the adverse health impact. The aim of this study is thus to use health statistics to assess the relative risk of asthma-related hospitalization for those living in close proximity to incineration facilities. We also examine differences in asthma risk related to age demographics. The spatial relationship between incineration facilities and asthma-related hospital admissions in Seoul is analyzed for the period of 2009–2011 using the Rapid Inquiry Facility (RIF) and SaTScan software. The relative risk of asthma-related hospitalization decreased with increasing distance from incinerators, but increased among those living within a 2-km radius. The relative risks of asthma-related hospitalization were 1.13 (95% confidence interval (CI): 1.10–1.17), 1.12 (95% CI: 1.08–1.17), and 1.18 (95% CI: 1.10–1.27) for all ages, those aged below 15 years, and those aged 65 years and older, respectively. This study is the first to observe an increased risk of asthma-related hospitalization in relation to a person’s distance from an incinerator in Seoul, Korea. It is clear that asthma should be considered an adverse health outcome during health impact assessments of incineration plants.

## 1. Introduction

The management of solid waste is a complex issue in many countries. While an increasing volume of solid waste is now being recycled, incineration is among the most commonly used technologies for solid waste disposal. In Seoul, the rates of incineration of municipal solid waste (MSW) increased rapidly from 2% in 1996 to 27% in 2012. Incineration of solid waste generates pollutants in the atmosphere (SO2, NO2, CO, fine and ultra-fine particulate, polycyclic aromatic hydrocarbons), metals, acids, and polychlorinated products (polychlorinated biphenyl (PCB), dioxins, furans) [1]. The emission of environmental pollutants from modern incinerators has been significantly decreasing with improved combustion practices and the use of air pollution control techniques albeit in small quantities and at very low levels. There is still great public concern over the adverse impact of incinerator emissions on human health [2]. There is often public opposition to the construction of new incinerators in local neighborhoods [3,4].

In response to these concerns, several studies focusing on health impact assessment have been conducted over the last decade [5,6]. Health effects associated with exposure to incinerator emissions are known to include increased risk of a range of cancers, respiratory symptoms, and congenital malformation [7,8]. While previous studies have focused more on cancer, even though the results have been controversial or inconclusive, few studies have focused on the respiratory symptoms. Among respiratory symptoms, asthma is considered a major cause of disability, health resource utilization, and poor quality of life worldwide. While the global prevalence of doctor-diagnosed asthma has been estimated at 4.3% [9], the prevalence of clinical asthma in Korea has been estimated at 3.9% (ranging from 2% to 13%). Furthermore, asthma is the 26th most frequently reported disease and is covered by national health insurance benefits [10]. Globally, asthma prevalence rates have increased over the last three decades, with similar trends witnessed in South Korea [11]. Considering that air pollutants, including ozone, SO2, NO2, PM10, and PM2.5, are well known as environmental triggers of asthma [12,13], it is reasonable to question the potential link between asthma and proximity to an incinerator.

Therefore, the aim of this study was to use health statistics to assess the relative risk of asthma-related hospitalization for people living in close proximity to incineration facilities. We also examined whether there were differences in asthma risk depending on age demographics. We used software designed to analyze the Spatial, Temporal, and Space-time Scan Statistics (SaTScan, https://www.satscan.org/) to identify geographical clusters of asthma-related hospitalization, while also using the Rapid Inquiry Facility (RIF) to estimate the relative risk of asthma-related hospitalization depending on the proximity of the incinerators in Seoul. The SaTScan software was developed by Martin Kulldorff together with Information Management Services Inc. and the RIF was developed for spatial epidemiological analysis, integrating both health outcomes and environmental data. This study provides findings in the assessment of the health impact of incineration plants and may help to develop public health policies designed to solve concerns regarding health and environmental hazards.

## 2. Materials and Methods

### 2.1. Study Area and Incinerators

The total area of Seoul is 605.2 km^2^, which accounts for 0.6% of the total area of the country. Seoul comprises 25 administrative districts (gus) and 424 sub-districts (dongs). As one of the most densely populated metropolises in the world, Seoul has a population of ca. 10 million and a population density of ca. 17,000 people per square kilometer, which is almost twice that of New York City.

**Figure 1:**
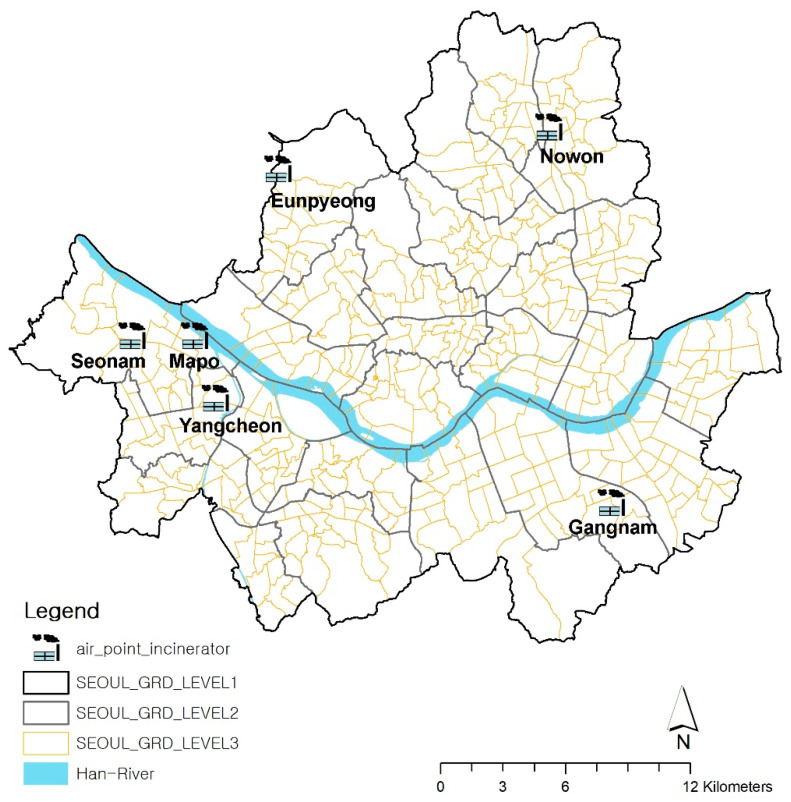
Location of Incineration Facilities, Seoul.

In Seoul, over 10,000 tons of MSW are produced daily. While approximately 65% of MSW was recycled in 2012, the percentage of MSW that underwent incineration increased from 2% in 1996 to 27% by 2012. A total of 12 incinerators at six MSW incineration plants were installed from 1996 to 2008. The locations of these six incineration facilities are mapped in Figure 1. The locations of the incinerators were used by converting the addresses provided by the Seoul Metropolitan Government into spatial data by geocoding based on the TM (Transverse Mercator) coordinate system. All six facilities dispose of household waste, and the quantities of waste depend on size. Eunpyeong incinerator is the smallest and handles up to 48 tons of waste per day, while Seonam incinerator processes up to 150 tons a day. Mapo incinerator can handle up to 750 tons while Yangcheon facility can handle up to 400 tons of waste per day. Nowon incineration facility can treat 800 tons of waste per day. Gangnam incinerator is the largest, which can handle up to 900 tons of waste per day.

### 2.2. Data

The spatial relationship between Seoul’s incineration facilities and its asthma-related admissions from 2009 to 2011 was analyzed in this study. For quantitative analysis, a database was constructed including regional geographical data, demographical data, health impact data, and environmental assessment data. Spatial analysis requires the use of the study area’s geographic data. In this study, data on the 424 sub-districts (dongs) of Seoul were extracted using shape files of the administrative boundaries in 2010, as provided by the Korea National Statistical Office.

Data regarding emission facilities are managed through the Stack Emission Management System (SEMS), which is overseen by the National Institute of Environment Research in Korea. Air emission facilities are categorized into five types, depending on the volume of emissions. In Seoul, there are a total of 19 air emission facilities. Of these, 13 are categorized as type 1 facilities, for which the extent of air pollutant emissions was greater than 80 tons per year as of 2010. Type 1 facilities are those that produce the highest emission levels. The six incineration facilities, which we selected for this study are all categorized as type 1, accounted for 68% of the total emissions in Seoul.

Asthma-related admission data were collected from the Korea National Health Insurance Claims Database of the National Health Insurance Service for the relevant period from 2009 to 2011. The International Classification of Disease (10th edition) was used as the diagnostic coding system. Only cases resulting in hospitalization were extracted from all the asthma (J45-J46) cases. Participants were divided into three groups according to age (under 15 years, 15 to 64 years, and 65 years and older), which were then analyzed by residential area and gender.

In order to identify the population characteristics by each sub-district (dong), resident registration data were collected from the Korea National Statistical Office from 2009 to 2011. Population figures according to gender and age, relevant to the area in question, were used for the investigation.

### 2.3. Disease Mapping and Risk Analysis

We employed three methods of analysis in different stages in conducting this study: disease mapping, cluster analysis, and risk analysis. The RIF was used for disease mapping and risk analysis, while cluster analysis was performed using the SaTScan.

Aylin et al. [14] developed the RIF that is embedded in ArcGIS 9.3 to analyze the point source pollution risk in England. The RIF supports disease mapping, which visualizes the mortality and morbidity of a given area [15]. Furthermore, it can identify the increased risk area of the disease, suggesting its geographical distribution, which can then be applied as a scientific basis in policy-making and resource distribution [16].

As the first step for the analyses, we calculated the ISRR (indirectly standardized relative risk) of the asthma-related admissions by sub-divisions (dongs) across Seoul in the RIF for disease mapping. ISRR is often used as an index for the risk of disease in epidemiological studies. ISRR projects the gender- and age-specific rates of the reference population onto the age and gender structure of the study population. The equation is as follows:

$$ISRR_{i}=\;\frac{O_{i}}{E_{i}}$$

In the equation, *ISRR_i_* is the indirectly standardized relative risk, *O_i_* is the observed value of the region *i*, and *E_i_* is the predicted value of the region *i*. $\;E_{i}=\sum_{j}N_{ij}r_{j}^{*}$, where *N_ij_* is the number of people at risk in the population at *j* level of age and gender in *i* region and r_j_^*^ is the specific disease rate at *j* level of age and gender of the standard region [15].

The disease map describes the spatial distribution of the relative risk of asthma-related admissions by region and age group across Seoul. The ISRR calculated in RIF is able to link with SaTScan to search for disease clusters.

Cluster detection was performed using SaTScan with a Poisson model [www.satscan.org]. Statistical cluster areas of hotspots were mapped based on SaTScan’s likelihood ratio [17]. This study used SaTScan’s Poisson model, and the likelihood ratio of the Poisson model is as follows:

(1) $$\gamma ={\left(\frac{c}{E\left[c\right]}\right)}^{c}{\left(\frac{C-c}{C-E\left[c\right]}\right)}^{C-c}I()$$

Here, *C* is the entire value and *c* is the observed value, *E[c]* is the predicted value of the observed value, and *I*() is the index factor. The clustered areas in which an increased incidence across Seoul was observed were compared with the locations of the incinerators, providing indirect implications regarding the health impact of hazardous facilities.

In terms of investigating the statistical relationship between proximity to the incinerators and the relative risk of asthma-related admissions, risk analysis was performed in the RIF. It is important to determine the buffer distance from the incineration facility in order to determine the correct neighborhood for analysis. In a previous study [18], which was performed on the air diffusion modelling of a municipal waste incineration facility, the air quality evaluation range had a radius of 5 km for the incineration facility, while the maximum ground level concentration of air pollution was found in areas within a 2-km radius of the facility. Based on these existing empirical results, we analyzed the relative risk of asthma-related admissions for the sub-districts located within buffer zones at distances of 2 km and 2–5 km from locations of the incineration facilities. Sub-divisions (dongs) entirely falling within the buffer area, as well as sub-divisions (dongs) with more than 50% of their area in the buffer, were included in the analysis. The buffer zone within 2 km of the incinerators includes 49 sub-divisions (dongs), 12% of the 424 sub-divisions in Seoul, and the 2–5 km buffer zone contains 138 sub-divisions, 33% of the total. The corresponding incident rate and the ISRR (indirectly standardized relative risk) were recalculated according to the buffer zone using the RIF. The risk analysis by buffer utilized the RIF. Eight countries (Britain, Denmark, Finland, Ireland, Italy, Spain, Sweden, and Netherlands) have used the RIF, developed in 1999, for the European Health and Environment Information System (EUROHEIS) project, and the US Centers for Disease Control and Prevention (CDC) National Environmental Public Health Tracking (EPHT) Network has utilized RIF. The validity and reliability were improved as an analytical tool through various projects and research conducted by numerous counties [19].

## 3. Results

### 3.1. Characteristics of the Study Area

The basic health impact statistics and demographical data in Seoul during 2009–2011 are described in Table 1. The population of Seoul was 9,794,000 as of 2010, while the average population of a dong, during 2009–2011, was 24,200. Of these, an average of 1693 were children, while 2345 were over 65 years old. During 2009–2011, a total of 61 people per dong were hospitalized due to asthma. Of these, 34 were children and 14 were seniors.

### 3.2. Asthma-Related Hospitalization Mapping and Cluster Analysis by Age Group in Seoul

Before investigating the spatial trends of the asthma-related hospitalizations by region, we simply compared the age- and gender-specific ISRRs for the entire study population (Table 2). The study population comprised the total population of Seoul, as of 2009–2011, divided into whole group, children group, and senior group. The ISRR of asthma-related admissions was 0.99 (95% confidence interval (CI) 0.97–1.01) for the whole group, 0.99 (95% CI: 0.97–1.00) for the children group, and 0.98 (95% CI: 0.96–1.01) for the senior group. There were no significant differences in the ISRR values for the groups analyzed by gender and age in the overall area of Seoul.

**Figure 2:**
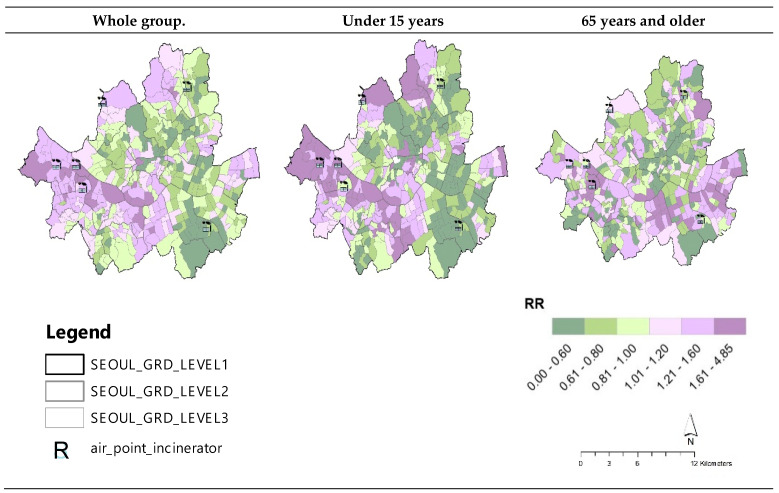
ISRR of Asthma-related Admissions by Age for each dong, Seoul (2009–2011).

The disease map of the ISRR for asthma-related admissions by age is described in Figure 2. The risk ratio of each dong in Seoul was investigated for the whole, children, and senior groups. There was no significant difference between the ages in the total population of Seoul. However, there were significant differences between different neighborhoods and age groups when the population was divided by sub-districts. The results showed that 22 dongs (sub-districts) across all ages, 59 dongs in the children group, and 52 dongs in the senior group had an ISRR of 1.61 or more, which represents a relatively high-level risk of asthma-related admissions. In particular, there was an increased ISRR for the whole group and children group in the west (Gangseo district) and for seniors in some sub-districts in western (Gangseo district) and southeastern (Gangnam district) Seoul.

**Figure 3:**
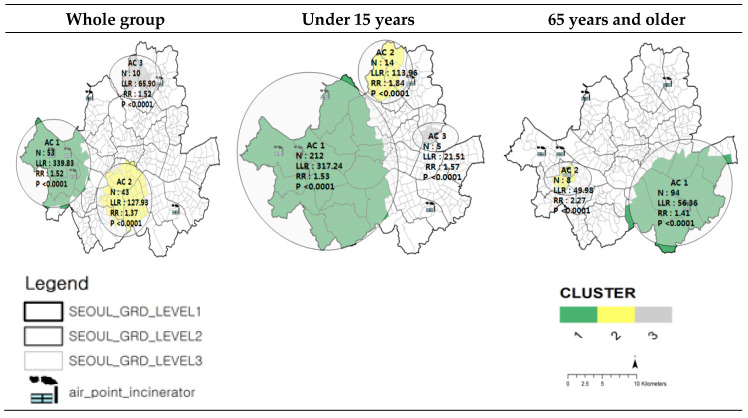
Clustered Areas of Asthma-related Admission by Age, Seoul (2009–2011).

Clusters with increased asthma-related admission rates during 2009–2011 were explored using SaTScan. The number of clustered areas as hotspots of asthma-related admission, likelihood ratios, and ISRR values from 2009 to 2011 are shown in Figure 3. The clustered area was decided on according to the differences in the likelihood ratio. Asthma cluster (AC) 1 had an increased incidence of asthma-related admissions with a maximum likelihood ratio. AC 1 comprised 53 sub-districts (dongs) in western Seoul (Gangseo gu) for the whole group, 232 sub-districts (including those of the whole group) for the children group, and 94 sub-districts to the southeast of Seoul (Gangnam gu) for the senior group. The whole group, children group, and senior group clusters were all statistically significant. When overlaying locations of incinerators on the hotspots indicating AC 1, the hotspots in both the whole age and children groups seemed highly relevant to the location of the three incinerators to the west of Seoul (Gangseo-gu). The hotspot for the senior group was also likely to be associated with the incinerator in Gangam-gu with a relatively large facility. The risk assessment was followed up to provide more quantitative analysis.

### 3.3. Risk Analysis of Asthma-Related Hospitalization

**Figure 4:**
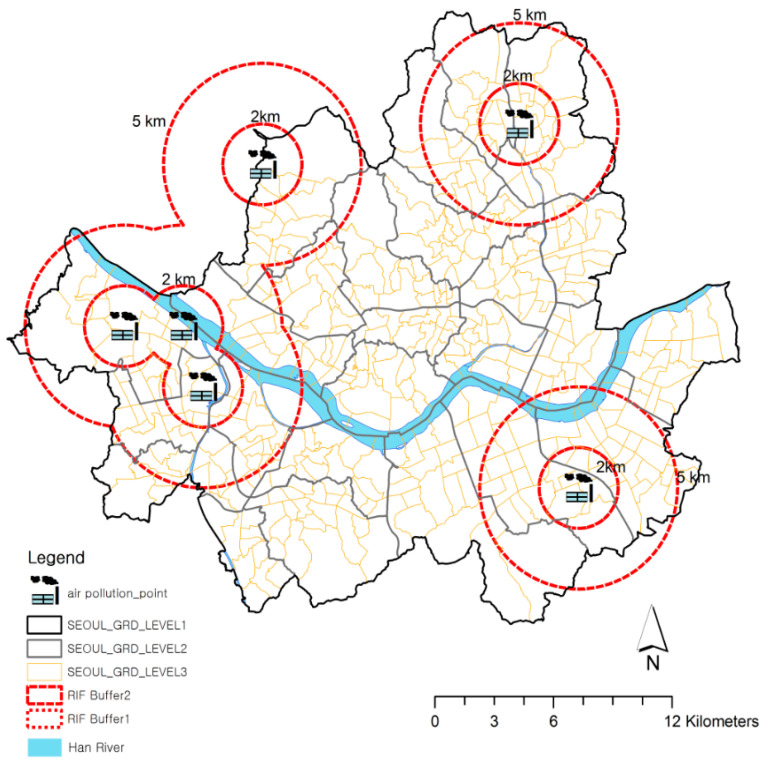
Buffer Zones from Incineration Facilities, Seoul.

Risk analyses of both the incineration facilities and asthma-related admissions were performed. The asthma-related hospitalization risks for the populations residing within 2 km and 2–5 km of the six incineration plants were analyzed, as shown in Figure 4. Analysis was performed using the RIF with data from 2009 to 2011. In the RIF, standardization by other covariates, such as socioeconomic status, is easily achieved in relation to gender and age. Thus, socio-economic data were adjusted utilizing the composition deprivation index (CDI), as suggested by Shin et al. [20], using unemployment, poverty, housing, labor, and social relationships as variables.

Table 3 describes the results of the risk analysis of asthma-related admissions in the regions located 0–2 km and 2–5 km from the incineration facilities. The asthma-related hospitalization risks within a 2 km and 2–5 km radius, shown in Table 3, were significantly higher than the reference values of the entire study population of Seoul (ISRR = 0.99) across all age groups, as indicated in Table 2.

As shown in Table 3, for the whole group, the risk of asthma-related admission was 1.13 (95% CI: 1.10–1.17) for people residing closer than 2 km from the incineration facilities, which was higher than the risk of those residing 2–5 km away, indicating an ISRR value of 1.00 (95% CI: 0.98–1.02). The ISRR significantly increased among those residing closer than 2 km to the incineration facilities, especially in both the children and senior groups. The value was highest for the senior group when analyzed by the separation distance from an incinerator. In particular, the male senior group living within 2 km of the incineration facilities had the highest risk value of 1.23 (95% CI: 1.10–1.38).

## 4. Discussion and Conclusions

Although the public is concerned about the negative impact of incinerator emissions on human respiratory conditions, little research has addressed the relationship between closeness to incineration facilities and the risk of asthma in Korea. This study was designed to identify the spatial relationship between incineration facilities and their health effects in Seoul from 2009–2011. We observed the spatial distribution of asthma-related admissions and disease clusters, which were hotspots with an increased asthma-related admission risk, and investigated these trends. Using the RIF, the specific asthma-related admission risks of those residing within 2 km and between 2 km and 5 km of the incineration facilities were analyzed.

Across the entire study population, there was no significant difference in terms of IRRS values among all age groups or the senior and children groups. However, when the data were disaggregated into sub-districts, disease maps of age-specific asthma admissions revealed significant spatial differences in the relative risks of different sub-districts. This indicated that the hotspots of asthma-related hospital admission were highly related to the location of incinerators. These results also mean that spatial distribution analysis can produce more meaningful results than showing the entire area in a single quantitative value. To find spatial clusters with a high risk of asthma-related admissions, this study employed SaTScan. Researchers in other countries [21,22] have been conducting disease monitoring using SaTScan, while only limited studies have been performed in Korea. We found hotspots with relatively high risks of asthma admissions by age group in Seoul, and the results showed that spatial analysis using SaTScan is also useful in a Korean context.

To achieve the main research objectives, this study analyzed the risk of asthma-related admission based on the separation distance from incineration facilities using the RIF. In the risk analysis using the RIF, the most interesting finding was that the risk of asthma-related admissions in areas adjacent to the incineration facilities (0–2 km) across the whole group, children group, and senior group were significantly higher than the reference values of the entire study population of Seoul (ISRR = 0.99). The relative risks of asthma-related hospitalization were 1.13 (95% confidence interval (CI): 1.10–1.17), 1.12 (95% CI: 1.08–1.17), and 1.18 (95% CI: 1.10–1.27) for all ages, those aged below 15 years, and those aged 65 years and older, respectively. Interestingly, the risk ratio of the senior group increased compared to that of the whole group and children group. The reason the elderly have a higher asthma risk than the children group seems to reflect the general trend. According to Chang [23], the prevalence of asthma in children was around 5–9%, but decreased to 3% in young adults, but asthma increased after 50 years, indicating a high prevalence rate of 6.8–12% among those 65 years or older.

This study makes some important theoretical contributions to the literature regarding the possible health effects of those residing close to incinerators. Existing studies have suggested the presence of three broad health concerns in association with residing close to an incinerator: cancer (all cancers, including that of larynx, lungs, esophagus, stomach, intestine, liver, kidney, bladder, and breast, as well as non-Hodgkin’s lymphoma and soft-tissue sarcoma), reproductive outcomes (infant deaths, congenital malformations, birth defects, and gestational age), and respiratory symptoms. While previous studies investigating the health effects of incinerators have focused predominantly on dioxins and cancer incidence [24,25], there have been difficulties in establishing causal relationships because of the characteristics of some types of cancer, such as low incidence rates and latent periods greater than 20 years. Therefore, identifying short-term health effects by utilizing health endpoints with high morbidities and excessive incidence rates or aggravations is required. In comparison, asthma is an appropriate surrogate marker for the health impact of incinerators, since it has high morbidity and requires continuous medical treatment. However, only a few studies have focused on the related respiratory effects.

Among studies focusing on respiratory effects in association with residing close to an incinerator, Hazucha et al. [26] and Lee and Shy [27] observed no difference in either the respiratory symptoms or pulmonary function between the populations living close to and far away from the incinerators. Gray et al. [28] also examined the frequency of occurrence of respiratory symptoms in children living near two sludge-burning incinerators, finding no differences in lung function, asthma prevalence, or atopy prevalence between the study and control populations. Ranzi et al. [29] evaluated the health impact of emissions from two incinerators in a pilot cohort study. They found that the mortality and morbidity experience due to respiratory diseases of the whole cohort did not differ from the regional population. Porta et al. [8], meanwhile, concluded that the association between incinerators and respiratory symptoms was inconclusive considering the uncertainty and residual confounding. Unlike the authors of other studies, Miyake et al. [30] suggested that the proximity of schools to municipal waste incinerators may be associated with an increased prevalence of wheezing, with an adjusted odds ratio of 1.08 (95% CI: 1.01–1.15). From the literature review, the association between the proximity to incinerators and respiratory symptoms was inconclusive.

In the midst of the discrepancies in previous studies, this study is meaningful in that it adds to the literature focused on the spatially significant relationship between proximity to incinerators and asthma-related risk. This study is the first to observe an increased risk of asthma-related hospitalization in relation to a person’s distance from an incinerator in Korea. Although there are uncertainties surrounding the estimated excess risk, mainly due to the less-sophisticated exposure assessment approach used in the analysis, it is clear that asthma should be considered an adverse health outcome during health impact assessments of incineration plants, which were not seriously considered in current assessments. The qualitative evaluation methodology and the results pertaining to this relationship may serve as a basis for environmental policymaking and the identification of vulnerable areas in the future.

In addition, this study confirmed that the RIF can be used to rapidly conduct ecological, environmental, and epidemiological analyses, including risk assessments and disease mapping. This approach builds on a method previously used by Beale et al. [15], who applied epidemiological, statistical, and geoinformatical methods in spatial epidemiology. Likewise, Holowaty et al. [31] used the RIF in the performance of spatial analyses for the determination of geographical changes in cancer incidences at Wellington-Dufferin–Guelph Public Health, Ontario, Canada. The standardized incidence ratio of cancer showed various spatial distribution forms. Similarly, Beale et al. [15] set the RIF in the application of statistical packages linked to it, such as SaTScan.

For elaborate risk analysis, the composition deprivation index (CDI) was taken into account as a potentially confounding factor in this study. Cesaroni et al. [32] found that the rate of lifetime asthma-related hospitalization was higher in the lowest income group, using individual and small area indicators of socioeconomic status (odd ratio 6.84 (95% CI: 2.1–22.5)). The approach corroborates the methods used by Shin et al. [20], who developed the CDI (Composite Deprivation Index) based on socioeconomic exclusion including the following five dimensions: unemployment, poverty, housing, labor, and social network.

Although this study provides a greater understanding of the health impact of incineration facilities, it also has some limitations that need to be considered. First, the relative risk was calculated based only on the separation distance from incineration facilities and not on the relationship between air pollution emissions and health effects. Having reviewed 41 studies on incinerators, Cordioli et al. [6] suggested an optimal utilization of pollution dispersion models in future studies. Of the reviewed 41 studies, 19 used linear distance as a measure of exposure to incinerators. Second, due to the limitations of data, the study did not adjust for traffic pollution, although traffic is among the major causes of air pollution in Seoul, as well as other air pollution sources such as factories and other land uses relating to air pollution. Thus, further research is needed to consider other covariates that were not included in this research to produce more precise statistical results. Although the buffer zone distance from incinerators was decided based on a previous study that performed the air diffusion modelling of a waste incinerator, the zone did not fit the real emission area. Due to this, the risk may have some potential error. Finally, while the disease risk may vary depending on the characteristics of different incineration facilities such as quantity and type of waste, this study did not consider these issues. Further research needs to focus on each facility and calculate the individual risks of each facility.

## Figures and Tables

**Table 1:** Basic Statistics of the Population and Asthma-related Admissions per dong in Seoul.

	Percentiles					Mean	Std. Deviation
	Min	25	Median	75	Max		
Population number (2009–2011, average)							
Whole group	1218	18,500	23,700	30,500	51,631	24,200	8882
Children group (under 15 years)	56	1170	1639	2147	4572	1693	785
Senior group (65 years and older)	141	1750	2270	2812	5240	2345	859
Asthma-related admissions (2009–2011, accumulated)							
Whole group	2	37	56	81.50	211	60.85	34.00
Children group (under 15 years)	1	17	28	47	144	33.84	22.54
Senior group (65 years and older)	1	10	13	17	19	13.95	6.88

**Table 2:** Age-specific and gender-specific indirectly standardized relative risk (ISRR) in Seoul.

Age/Gender		ISRR		
		Observed Value	Expected Value	ISRR (95% Confidence Interval)
Whole age group	Male	12,969	13,093.51	0.99 (0.97–1.01)
	Female	12,649	12,803.44	0.99 (0.97–1.01)
	Total	25,618	25,896.96	0.99 (0.98–1.00)
Children group (under 15 years)	Male	8108	8213.42	0.99 (0.97–1.01)
	Female	5977	6055.49	0.99 (0.96–1.01)
	Total	14,085	14,268.91	0.99 (0.97–1.00)
Senior group (65 years and older)	Male	2289	2305.73	0.99 (0.95–1.03)
	Female	3044	3113.83	0.98 (0.94–1.01)
	Total	5333	5419.55	0.98 (0.96–1.01)

**Table 3:** ISRR of Asthma-related Admissions by the Distance from the Facility and Age Group, Seoul.

Age/Gender	0~2 km from the Incineration Facility			2~5 km from the Incineration Facility		
	Observed	Expected	ISRR (95% Confidence Interval)	Observed	Expected	ISRR (95% Confidence Interval)
Whole group						
Male	2051	1806.97	1.14 (1.09–1.19)	4712	4640.94	1.02 (0.88–1.04)
Female	1979	1753.64	1.13 (1.08–1.18)	4425	4482.29	0.99 (0.96–1.02)
Total	4030	3560.61	1.13 (1.10–1.17)	9137	9123.22	1.00 (0.98–1.02)
Under 15 years						
Male	1350	1217.02	1.11 (1.05–1.17)	2974	2955.28	1.01 (0.97–1.04)
Female	1014	884.95	1.15 (1.08–1.22)	2121	2176.3	0.97 (0.93–1.02)
Total	2364	2101.96	1.12 (1.08–1.17)	5095	5131.58	0.99 (0.97–1.02)
65 years and older						
Male	315	255.33	1.23 (1.10–1.38)	820	799.41	1.03 (0.96–1.10)
Female	441	383.7	1.15 (1.05–1.26)	1058	1048.35	1.10 (0.95–1.07)
Total	756	639.02	1.18 (1.10–1.27)	1878	1847.76	1.02 (0.97–1.06)
